# B-1 cells and B-1 cell precursors prompt different responses to Wnt signaling

**DOI:** 10.1371/journal.pone.0199332

**Published:** 2018-06-21

**Authors:** Lika Osugui, Jolanda J. de Roo, Vivian Cristina de Oliveira, Ana Clara Pires Sodré, Frank J. T. Staal, Ana Flavia Popi

**Affiliations:** 1 Departamento de Microbiologia, Imunologia e Parasitologia, Escola Paulista de Medicina, Universidade Federal de São Paulo, São Paulo, Brazil; 2 Department of Immunohematology and Blood Transfusion, Leiden University Medical Center, Leiden, The Netherlands; National Cancer Center, JAPAN

## Abstract

Recently several studies demonstrated a role for the Wnt pathway in lymphocyte development and self-renewal of hematopoietic stem cells (HSCs). B-1 cells constitute a separate lineage of B lymphocytes, originating during fetal hematopoiesis, expressing lymphoid and myeloid markers and possessing self-renewal ability, similar to early hematopoietic progenitors and HSCs. A plethora of studies have shown an important role for the evolutionary conserved Wnt pathway in the biology of HSCs and T lymphocyte development. Our previous data demonstrated abundant expression of Wnt pathway components by B-1 cells, including Wnt ligands and receptors. Here we report that the canonical Wnt pathway is activated in B-1 cell precursors, but not in mature B-1 cells. However, both B-1 precursors and B-1 cells are able to respond to Wnt ligands *in vitro*. Canonical Wnt activity promotes proliferation of B-1 cells, while non-canonical Wnt signals induce the expansion of B-1 precursors. Interestingly, using a co-culture system with OP9 cells, Wnt3a stimulus supported the generation of B-1a cells. Taking together, these results indicate that B-1 cells and their progenitors are differentially responsive to Wnt ligands, and that the balance of activation of canonical and non-canonical Wnt signaling may regulate the maintenance and differentiation of different B-1 cell subsets.

## Introduction

B-1 cells constitute a subpopulation of B cells, mostly found in the peritoneal cavity and rarely in the spleen. Besides possessing B lineage markers (CD19^HI^IgM^HI^IgD^lo^), their expression is quantitatively different from conventional B cells (CD19^+^IgM^lo^IgD^HI^), and B-1 cells also express the myeloid marker CD11b. Further, B-1 cells are subdivided in two subtypes: B-1a and B-1b owing to CD5 expression in the former. This heterogeneity is also seen in the progenitor populations of these cells. Ample evidence supports the existence of distinct ontogenic lineages, in which B-1a cells are generated largely from fetal liver progenitors, and maintained in adult life mostly by self-renewal, while B-1b cells, albeit having self-renewal, can be generated de-novo via HSCs located in the BM [1–6].

Several molecules are involved in promoting HSC differentiation. Among them Wnt proteins play an important role in controlling cell fate, proliferation and asymmetric cell division [7, 8]. Wnt proteins are a family of highly conserved glycoproteins encoded by 19 different genes. In the canonical way, the binding of one of Wnt ligands (such as Wnt1, Wnt3a and Wnt8) to a Fzd family receptor and LRP5/6 co-receptor activates an intracellular signaling pathway, leading to the inactivation of the β-catenin destruction complex. This complex is formed by: tumour-suppressor gene products axis inhibitor-1 and 2 (AXIN) and adenomatous polyposis coli (APC), the serine/ threonine kinases casein kinase 1 (CK1) and glycogen synthase kinase 3β (GSK3β). After inactivation of the destruction complex, β-catenin accumulates in the cytoplasm and consequently is translocated to the nucleus, where it activates TCF (T cell factor)-LEF (Lymphocyte enhancing factor) transcription factors. The other two less well-defined pathways are independent of β-catenin translocation and include the Wnt/Ca^2+^ pathway and the planar cell polarity (PCP) pathway [7, 9, 10].

During the differentiation of HSCs into committed precursors, the Wnt/β-catenin pathway activity is reduced [11], except for the T cell lineage [12, 13]. Several studies support that the canonical Wnt pathway regulates some aspects of B cell development (8,9,12,18). It was described that Lef1-deficient mice have a mild block in fetal, but not adult B lymphopoiesis (18). As reviewed by Staal and Clevers (8), the lower expression of TCF and LEF by mature B cells reflect that the activity of canonical Wnt pathway in mature B cells is very low. The low Wnt activity in mature B cells also has been reported using different in vivo Wnt reporter mice (12).

However, forced activation of β-catenin in the lymphoid precursors silences the expression of EBF and Pax-5, reversing their previous commitment to B lineage [14]. On the other hand, high levels of β-catenin in the myeloid precursors increase the expression of EBF, resulting in the generation of lymphocytes from these cells [15]. Taken together, these data reveal that Wnt/ β-catenin pathway helps regulate the development and commitment of the B cell lineage.

It has been also demonstrated that pro-B cells from LEF-1 deficient mice exhibit defects in cell proliferation and survival *in vitro* and *in vivo*. The addition of LiCl, Wnt3a conditioned medium or recombinant Wnt3a resulted in pro-B proliferation [11, 16, 17]. In spite of that, mice with β-catenin depletion specific in B cells have normal B cell development in bone marrow and periphery. Interestingly, the authors noted that B-1 cells in the peritoneal cavity of these mice were reduced, and the absolute number of B-1b cells was 50% lower than in wild type mice [18]. A previous report has demonstrated that blockage of Wnt pathway by quercetin induces apoptosis of B-1 cell *in vitro*. In this study, authors also demonstrated a reduction in the IL-6 levels in the presence of quercetin, which could be related to a decrease in B-1 cell proliferation and viability *in vitro* [19]. Thus, B-1 cells could be an interesting model to study the influence of Wnt pathway in lineage fidelity and commitment, since B-1 cells are self-renewing cells, which express both lymphoid and myeloid programs simultaneously, and also have the ability to differentiate into phagocytes *in vitro* and *in vivo* [20–22]

Based on these data, we investigated the activation of Wnt pathway in B-1 cells and also in the B-1 cell precursors aiming to elucidate the role of this pathway in the B-1 cell development.

## Material and methods

### Mice

C57BL/6 female mice, 8 weeks, were obtained from the Centro de Desenvolvimento de Modelos Experimentais para Medicina e Biologia (CEDEME) of the Universidade Federal de São Paulo (UNIFESP). Axin2^+/lacZ^ Wnt-reporter female mice, between 6–12 weeks and also two weeks old Axin2^+/lacZ^ and WT littermates were handled and euthanized, following the guidelines of Leiden University Medical Center (LUMC) Ethical Committee. All animals were maintained under pathogen free conditions. All procedures described here were approved by the Ethical Committee from UNIFESP (2012/712).

### Characterization of B-1 and B-1P cells

Cells from the peritoneal cavity or bone marrow from Axin2^+/lacZ^ Wnt-reporter mice and C57BL/6 wild type *(*WT) mice were used. The peritoneal cells were harvested by washing out the peritoneal cavity using RPMI1640 medium. The bone marrow cells were isolated from both femurs of each animal, and clear bones were crushed using a mortar and pestle. The crushed bones were rinsed with RPMI1640 medium, and the supernatant were collected and filter using a 40 μm cell strainer. After that, cells were counted and pre-stained with anti-CD16 CD32 mAb to block Fcγ RIII/II receptors and stained on ice for 30 min with the monoclonal antibodies against the following molecules: CD19, CD23, CD11b, CD5 to characterize B-1a (CD19^+^CD23^-^CD11b^+/-^CD5^+^) and B-1b (CD19^+^CD23^-^CD11b^+/-^CD5^-^) cells subsets from peritoneal cavity and with CD3e, CD4, CD11b, Gr-1, IgM, NK-1.1, Ter119, CD45R/B220, CD19 and CD93 (Early B—AA4.1) to characterize B-1 progenitors (B-1P - Lin^-^CD19^+^EarlyB^+^B220^lo/neg^) from bone marrow. B-1 cells and B-1 cell precursors were also stained with anti-Flt3 and IL7-R antibodies to determine the expression of these receptors. Cells were acquired using the BD FACSCanto™II flow cytometer and data were analyzed with FlowJo software (S1 and S2 Figs).

### Enrichment of B-1 and B-1P cells

B-1 cells population were cell sorted by BD FACSAria III from mice’s peritoneal cells. First, cells were collected and processed as described above. Two strategies were used to purify B-1 cells population: negative or positive selection. For the negative selection strategy, cells were stained with CD3 and CD23 antibodies. From the lymphocyte gate, a double negative population (CD3^-^CD23^-^) was sorted. After that, an aliquot of sorted cells was fully stained to confirm the B-1 cell purity. For the positive selection, CD19 and CD23 antibodies were used, and the CD19^+^CD23^-^ population from the lymphocyte gate was sorted. In all experiments the B-1 cell purity was around 95% after cell sorting.

Bone marrow was processed and stained as described above (item 2) and B-1P cells were enriched by cell sorting, using the following strategy: from lineage negative population (CD3e, CD4, CD11b, Gr-1, IgM, NK1.1, TER119) and CD93^+^, a CD19^+^B220^lo/neg^ population was sorted.

B-1 and B-1 cell precursor enriched population were obtained from pooled sorting cells from 5–7 mice, and were considered one biological sample. Each experiment was performed with 2–3 biological samples as indicated in each Fig.

### B-1 cell culture

Purified B-1 cells were cultivated in RPMI medium added with 10% fetal calf serum (FCS), 5x10^-5^ M 2-β-mercaptoethanol, 1 mM L-glutamine, 100 U mL^-1^ streptomycin, 100 μg mL^-1^ penicillin. When indicated in the text, recombinant Wnt3a and Wnt5 (100ng/ml) were added daily for 72 hours. In other experiments, IL7 was also added to the cultures. After this, B-1 cells were collected and submitted to the experiments protocols as described below.

### Gene expression analysis

RNA from purified B-1 cells and bone-marrow-derived total cells was isolated using Pure Link Kit RNA (Life Technologies). The cDNA was obtained using the Superscript III cDNA Synthesis (Life Technologies*)*. Expression levels of FZD receptors gene, *AXIN2*, *FLT3*, *IL7R* and *PAX-5* were assessed by real-time PCR using a FAST Sybr Green Reagent (Applied Biosystems) on an Applied Biosystems 7500 Fast Real-Time PCR System. The amplification efficiencies were determined by comparing the dilution series of reference and target genes from a reference cDNA template. The amplification efficiency was calculated using the following equation: E = 10^(−1/slope)^ − 1, in which E is the efficiency and slope is the value obtained by constructing standard curve. A validation was performed to evaluate if the efficiencies of the target and the reference gene were approximately equal (90% ≤ E ≤ 110%). If the target and the reference genes had comparable amplification efficiencies, relative quantification was determined according to the 2^−ΔΔCt^ or 2^−ΔCt^ method, as indicated in each Fig [23, 24]. Each reaction was carried out in triplicate using at least three biological samples. The sample used as normalizer was bone-marrow derived total or control B-1 cells, as indicated in each experiment.

### Proliferation analysis

After purification, B-1 cells and B-1 precursors were stained using 5 μM of Cell Proliferation Dye eFluor® 670, following the manufacture instructions and submitted to different cell culture conditions, as indicated in each experiment. The maximum of CFSE staining cells in time zero were considered to determine the region gate of non-proliferative cells. To measured the decay of fluorescence, which is not related to proliferation, B-1 cells or B-1 cell precursor cultured in a RPMI medium only was used. The decay of fluorescence in these samples was subtracted to the decay of fluorescence in the experimental groups to determine the gate region of fluorescence cells and also MFI.

### Canonical Wnt signaling evaluation

Cells from Axin2^+/lacZ^ Wnt-reporter mice were obtained as described above (item 2) and the Wnt signaling was evaluated by measurement of the β-galactosidase activity (lacZ), as previously described [25, 26]. Briefly, up to 5x10^6^ cells suspension was loaded with 2 mM of fluorescein di β-D-galactopyranoside (FDG, Molecular Probes) by hypotonic shock. After one minute, 10x volume of ice-cold medium was added to restore the isotonicity. The reaction was stopped two hours later by adding 1 mM of PETG (phenylethyl β-D-thiogalactopyranoside), following the surface markers staining. The cells were acquired using the BD FACSCanto™II flow cytometer and data were analyzed with FlowJo software. In all experiments cells from WT mice were submitted to the same treatment to determine negative and positive gates. In order to calculate the percentage or absolute number of FDG+ cells of each subset, FDG+ amount was subtracted from amount of FDG+ cells in the WT mice in order to correct differences in background staining.

### Co-culture of B-1P cells and Wnt-transduced OP9 cells

Purified B-1P cells were co-cultured on OP9 stromal cell lines transduced or not with Wnt constructs (OP9-WT, OP9-Wnt3a, OP9-Wnt5a), generated by Famili et al. [27], in 48 well plates, as previously described [1]. The OP9 layers (1.0x10^4^ cells per well) were seeded 24 hours before the test, with α-MEM + 20% FCS. B-1 progenitors were purified and resuspended in progenitor medium (RPMI 1640, 10% FCS, 5x10^-5^ M 2-β-mercaptoethanol, 1 mM L-glutamine, 100 U mL^-1^ streptomycin, 100 μg mL^-1^ penicillin, 20 ηg mL^-1^ IL-3, 20 ηg mL^-1^ IL-6, 20 ηg mL^-1^ SCF, 10 ηg mL^-1^ Flt-3 ligand and 10 ηg mL^-1^ IL-7), the OP9 medium was discarded and 3x10^3^ B-1P cells were cultured on the OP9 layers. The cultures were incubated at 37°C and 5% CO_2_. At the 9^th^ culture day, cells were counted and analyzed by flow cytometry.

## Results

### Wnt components expression by B-1 cells

**Fig 1:**
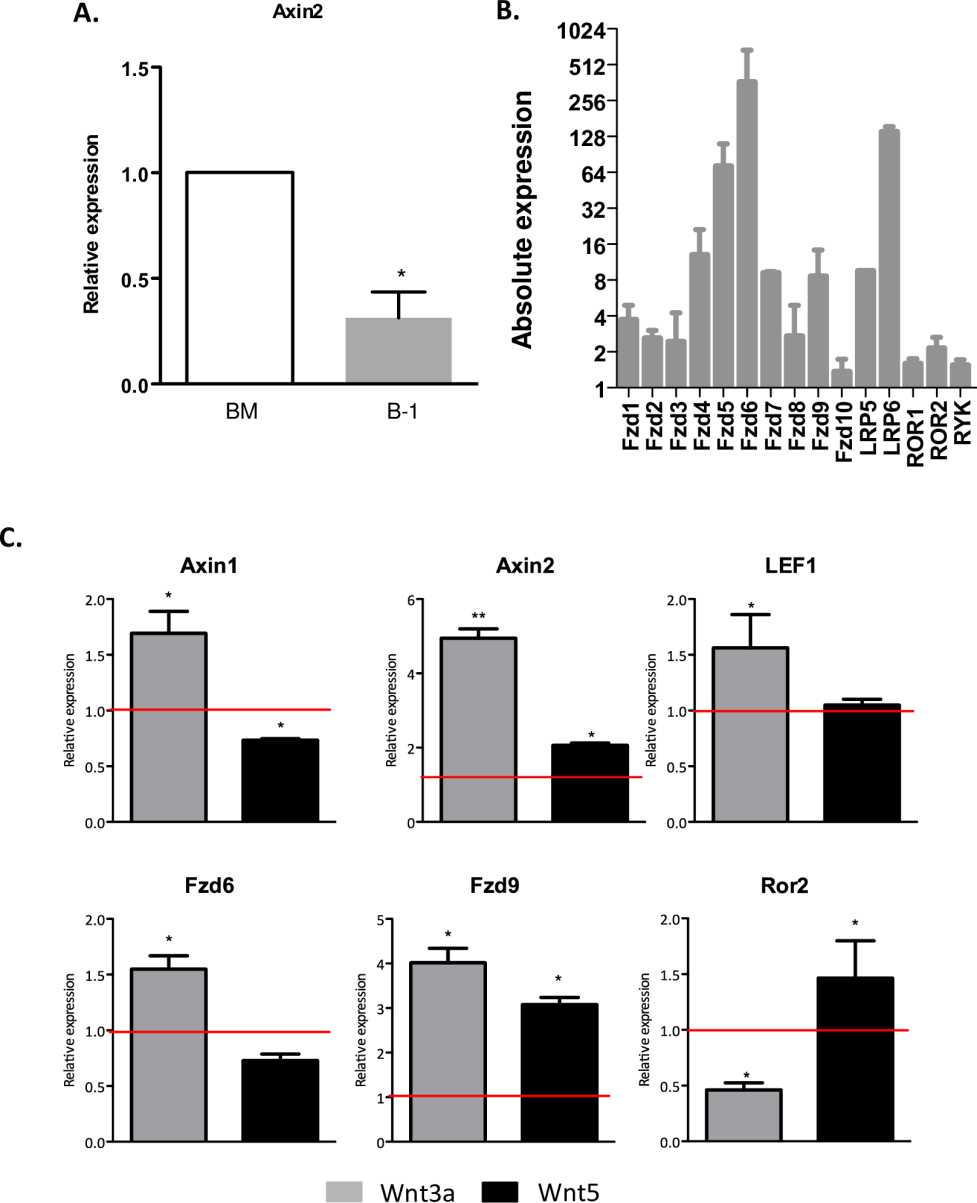
B-1 cells are responsive to Wnt ligands. A) Relative expression of *Axin2* by B-1 cells. Rplp0 gene was used as reference gene. Relative expression is 2^-ΔΔCt^, considering control bone marrow cells B-1 cells as a normalizer. Data from 3 biological samples per experiment, each one plated on triplicate. Data shown are representative of 2 experiments. *p<0,05 B) Expression of Wnt receptors, co-receptors and other Wnt target genes by purified B-1 cells determined using 2^-ΔCT^. Normalization was performed using Rplp0 as reference gene. Data from a representative of 3 experiments performed at triplicate of each biological sample (n = 3). C) Expression of Wnt target genes and receptors by B-1 cells stimulated in vitro by Wnt3a (100ng/ml) and Wnt5a (100ng/ml) daily for 3 days. Rplp0 gene was used as reference gene. Relative expression is 2^-ΔΔCt^, considering non-treated B-1 cells as a normalizer (red line). n = 3 biological sample per experiment, each one plated on triplicate. Data shown are representative of 2 experiments. *p<0,05 and **p<0,01 when indicated group were compared to non-treated cells.

In order to consider if B-1 cells were able to respond to Wnt signaling, we evaluated the expression of *AXIN2*, FZD receptors and other Wnt target genes. We first compared bone-marrow-derived total cells and purified peritoneal B-1 cells for expression of Wnt components. As expected, *AXIN2* levels were lower in B-1 cells than in bone-marrow derived total cells (Fig 1A). Despite of B-1 cells express all FZD receptors, *LRP5*, *LRP6*, *ROR1*, *ROR2* and *RYK*; we detected higher levels of expression of *FZD6* and *LRP6* (Fig 1B). Considering this, we could assume that B-1 cells are capable to respond to Wnt ligands.

In the next step, we investigated if B-1 cells could be responsive to Wnt ligands. Purified B-1 cells were stimulated *in vitro* with recombinant Wnt3a (100ng/ml) or Wnt5a (100ng/ml) proteins daily. After 72 hours we observed that the Wnt3a stimulus augmented the expression of *AXIN1*, *AXIN2*, *LEF1*, *FZD6*, *FZD9* and decreased the expression of *ROR2*. Wnt5a augmented the expression of *AXIN2*, *FZD9* and *ROR2* (Fig 1C). These data suggest that the presence of Wnt3a augmented the responsiveness of B-1 cells to canonical Wnt signaling, while Wnt5a augmented at least the expression of *ROR2*.

### Activation of Wnt signaling by WNT3a induces B-1 cell proliferation and increases expression of IL7R *in vitro*

**Fig 2:**
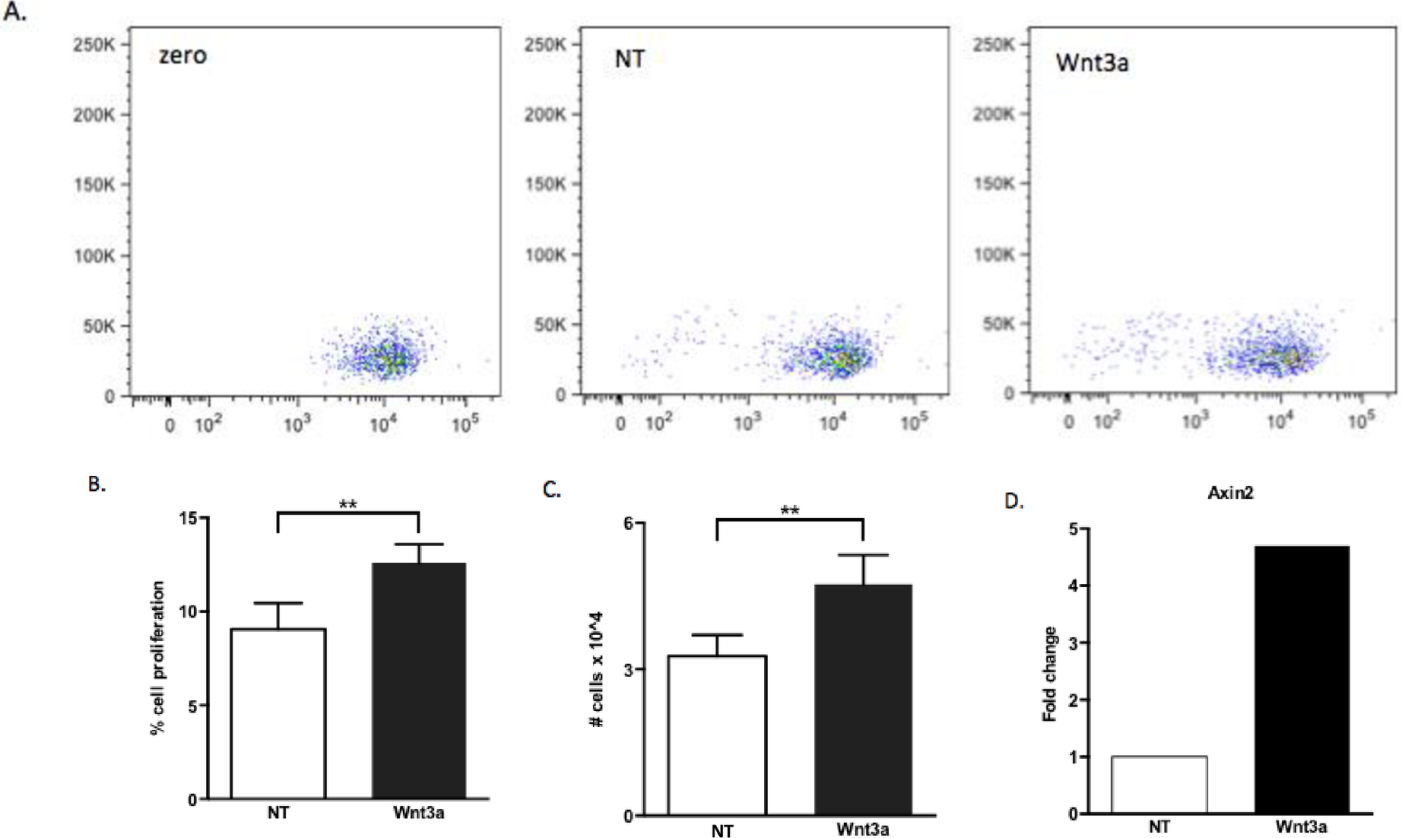
Wnt3a increases B-1 cell proliferation *in vitro*. Purified B-1 cells from C57BL/6 mice were were stimulated with recombinant Wnt3a protein (100ng/mL) was added daily. (A) Representative dot plots of proliferation of B-1 cells: time zero (zero), non- treated cells (NT) and in the presence of Wnt3a. After 72 hrs, percentage (B) and absolute number (C) of B-1 cells in proliferation were determined. The Wnt activation was evaluated by qPCR by Axin2 gene expression (D). Normalization was performed using Rplp0 as reference gene. Non-treated B-1 cells were used as normalizer sample. Relative expression was determined using 2^-ΔΔCT^. Data from a representative of 3 experiments performed at triplicate from 3 biological samples. **p≤0,001 (Mann-Whitney test).

Considering that the presence of Wnt ligands in the B-1 cell culture stimulated the expression of some genes that prompt B-1 cells to respond to them, our next step was to investigated if Wnt stimuli could modulate cell proliferation. We demonstrated that Wnt3a increased the proliferation of B-1 cells *in vitro* (Fig 2A, 2B and 2C) and also increased the expression of *AXIN2 (*Fig 2D), which demonstrates that the Wnt pathway is activated by this stimulus.

**Fig 3:**
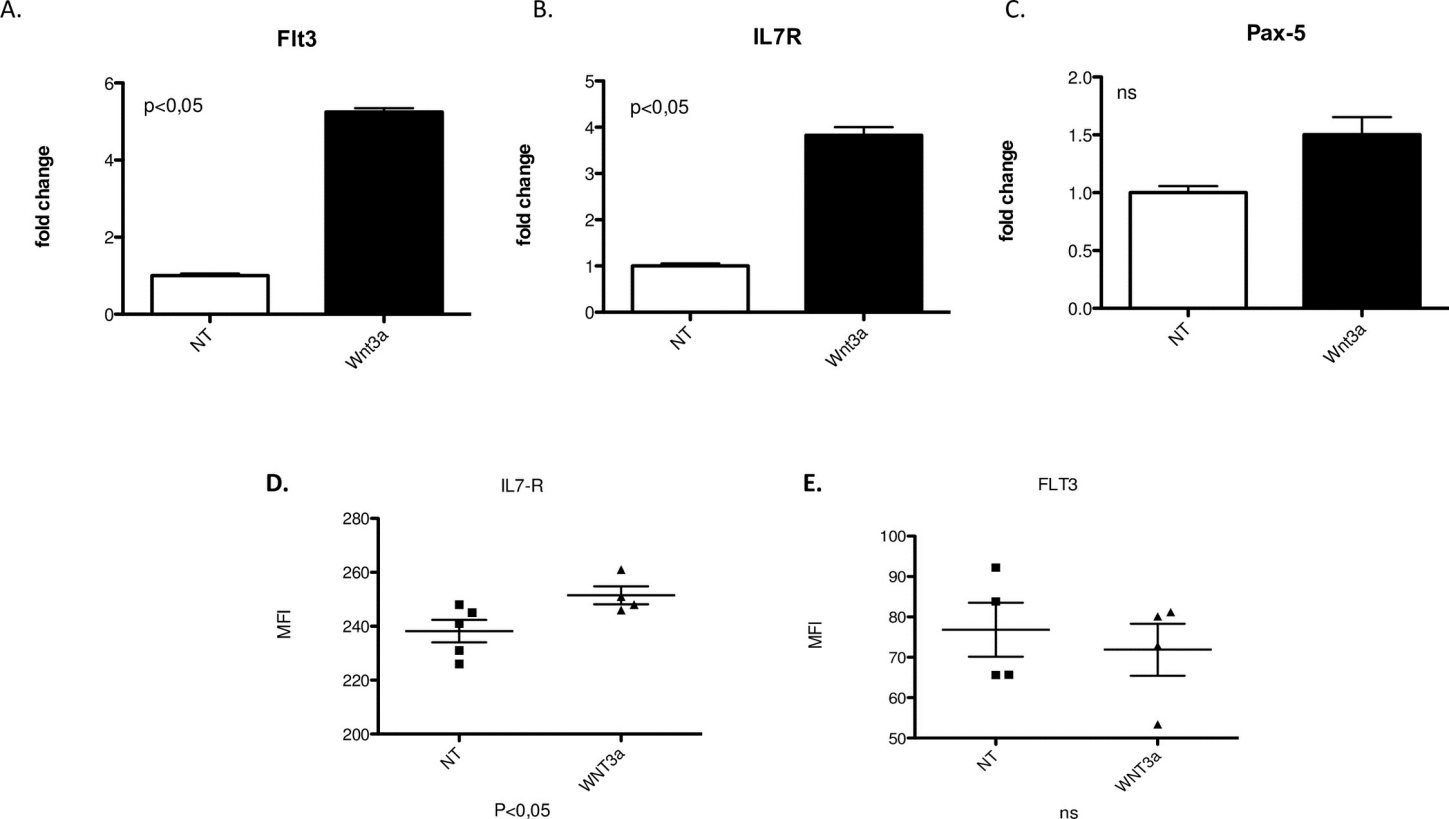
IL7R overexpression in Wnt3-treated B-1 cells. Purified B-1 cells from C57BL/6 mice were daily treated or not (NT) with 100 ng of Wnt3a (Wnt3a) recombinant. After 72 hrs, the relative expression of lymphoid transcription factors FLt3 (A), IL7R (B), Pax-5 (C) were calculated, using non-treated group (NT) as a normalized sample. Normalization was performed using Rplp0 as reference gene. Relative expression was determined using 2^-ΔΔCT^. Data from a representative of 2 experiments performed at triplicate. 3 biological samples were used in each experiment. *p≤0,01 and **p≤0,001 (Mann-Whitney test). D. MFI (Mean of Fluorescence Intensity) of IL7R expression by B-1 cells in the presence of Wnt3a or not (NT). Data from a representative of 2 experiments performed (n = 5). E. MFI (Mean of Fluorescence Intensity) of Flt3 expression by B-1 cells in the presence of Wnt3a or not (NT). Data from a representative of 2 experiments performed (n = 5).

Interestingly, stimulation with Wnt5a did not induce any change in B-1 cell viability or proliferation (*data not shown*). Additionally, Wnt3a augmented *FLT3* and *IL7R* gene expression in B-1 cells (Fig 3A and 3B), but did not modify *PAX5* expression (Fig 3C). At protein level we confirmed that Wnt3a induced an increase in the IL7-R expression by B-1 cells, but not Flt3 expression (Fig 3D and 3E).

### Wnt3a increases B-1 cell responsiveness to IL7

**Fig 4:**
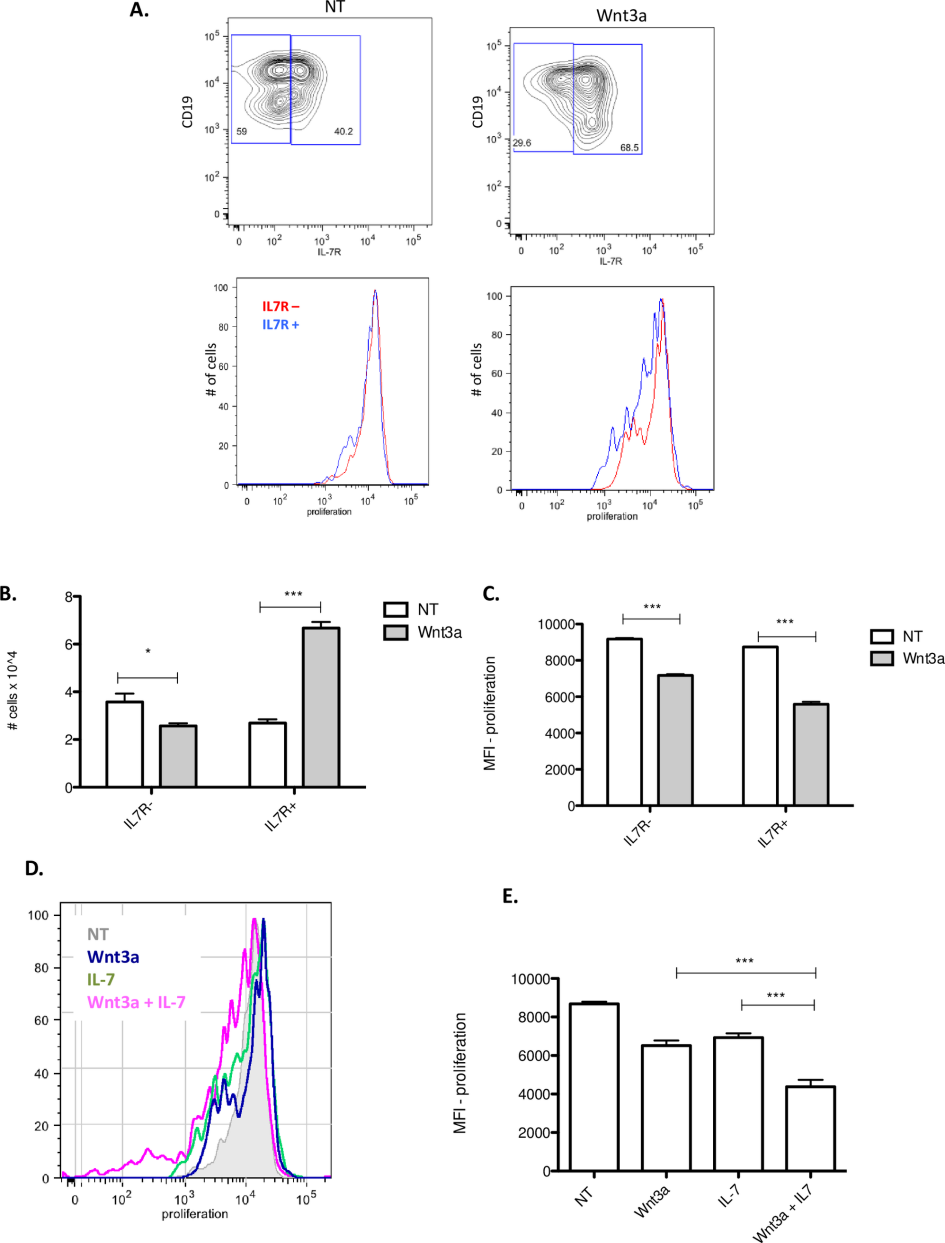
Wnt3a stimulation increment proliferation of B-1 cells in response of IL7. Purified B-1 cells from C57BL/6 mice were treated with Wnt3a (100ng/ml), IL-7 (50ng/ml) and Wnt3a+IL7 during 3 days. Non-treated cells (NT) were used as control group. A) Contour plots analysis of expression of CD19 and IL7R by non-treated B-1 cells or Wnt3a-treated B-1 cells. Histograms show the proliferation (CFSE decay) of IL7R^+^(blue) or IL7R^-^(red) B-1 cells in control group or Wnt3a group. The maximum of CFSE staining cells in time zero were considered to determine the region gate of non-proliferative cells. To measured the decay of fluorescence, which is not related to proliferation, B-1 cells cultured in a RPMI medium only was used. The decay of fluorescence in these samples was subtracted to the decay of fluorescence in the experimental groups to determine the gate region of fluorescence cells and also MFI. B) Absolute number of B-1 cells IL7R^-^ or IL7R^+^ cells from non-treated group (NT) or Wnt3a treated group (Wnt3a). C) Proliferation of IL7R^-^ or IL7R^+^ B-1 cells from non-treated group (NT) or Wnt3a treated group (Wnt3a) measured by decay in the MFI value. D) Histograms of CFSE decay (proliferation) of B-1 cells in the different groups: NT (non-treated), Wnt3a, IL-7, Wnt3a+IL7. E) Proliferation of B-1 cells was represented by MFI value in different conditions: NT (non-treated), Wnt3a, IL-7, Wnt3a+IL7. ***p<0,001, **p<0,01 and *p<0,05 (One way ANOVA). Data from a representative of 3 independent experiments performed in A,B and C and 2 independent experiments performed in D and E. Each experiment was performed using 3 biological samples.

Considering this, we decided to investigate if the increment in the proliferation in the presence of Wnt3a could be not a direct effect of Wnt ligand stimulus, but due to an induction of IL7-R expression. Corroborating this hypothesis, a higher number of IL7R^+^ cells were detected in Wnt3a treated group (Fig 4A) in comparison to control group (NT—non-treated). As observed in the Fig 4A, 68,5% of B-1 cells from the Wnt3a treated group expressed IL7R, while only 40% of B-1 cells are IL7R^+^ in control group (NT). Corroborating on this, the absolute number of IL7R^+^ B-1 cells are a approximately 6.67x10^4^ cells in Wnt3a group and almost 2.5 times less in control group (2.69 x10^4^ cells) (Fig 4B). Considering that the absolute number of B-1 cells in the culture after 72 hours of Wnt3a stimulus is higher than control group, we investigated the proliferation and viability of these cells. Wnt3a did not modify the cell viability in culture, however we observed that proliferation index is higher after this treatment. Furthermore, IL7R^+^ B-1 cells are more proliferative than IL7R^-^ B-1 cells, at least in the presence of Wnt3a (Fig 4A–lower panel and Fig 4C). It was observed that both IL7R^-^ and IL7R^+^ B-1 cells are more proliferative in the presence of Wnt3a in comparison to control group. Besides, in the Wnt3a conditions, IL7R^+^ cells are more proliferative than IL7R^-^ cells, as measured by the decay of CFSE fluorescence (Fig 4C).

To elucidate this, proliferation of B-1 cells were analyzed in the presence of Wnt3a only, IL-7 only or Wnt3a + IL-7. Expectedly, B-1 cell proliferation is enhanced by addition of IL-7 recombinant, as well as in addition of Wnt3a stimulus (Fig 4D and 4E). The addition of Wnt3a+IL7 promotes higher levels of proliferation, which suggests that increased IL7-R expression caused by Wnt3a could promote B-1 cell proliferation *in vitro* via IL7 signaling. Whether this increase is due to increased expression of IL7-R on all B1 cells or preferentially on cells already expressing IL7-R via an autocrine loop remains to be elucidated. Nevertheless, our current findings are mostly consistent with Wnt3a acting via the IL7/IL7R axis to induce B-1 cell proliferation.

### Wnt signaling is not activated in B-1 cells in vivo

**Fig 5:**
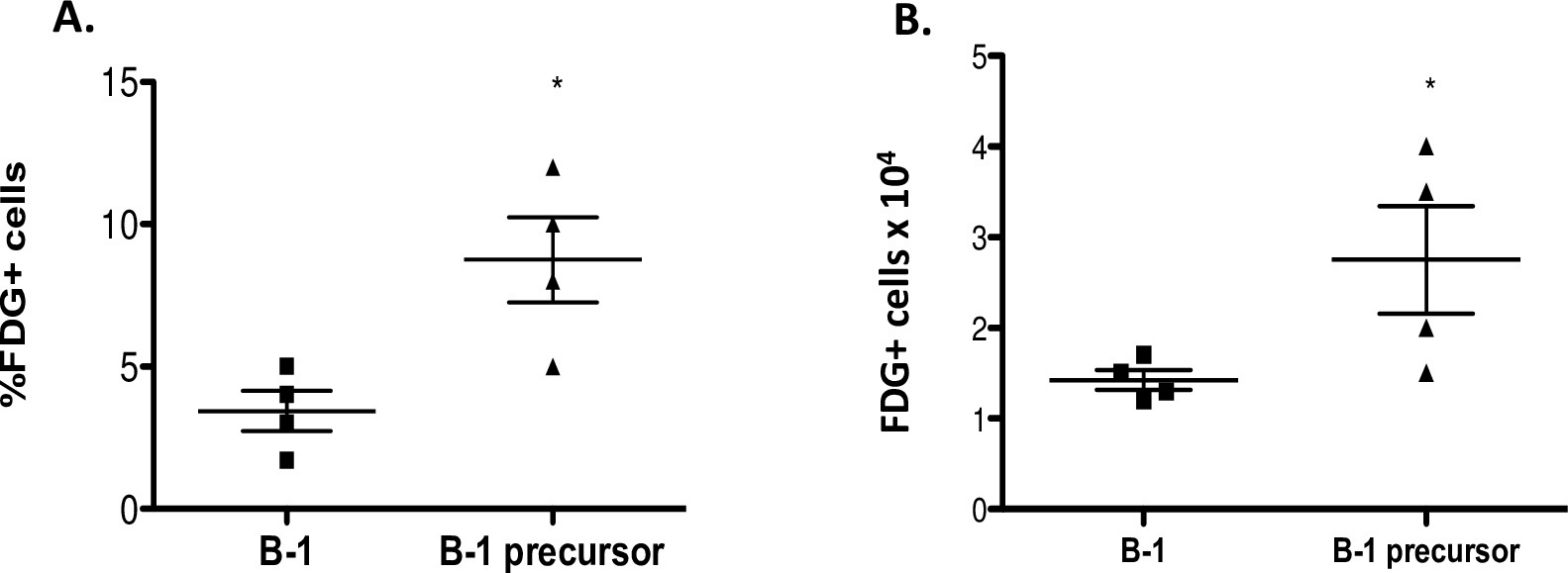
Canonical Wnt signaling is activated in B-1 precursors, but not in B-1 cells. B-1 progenitors from bone marrow and peritoneal B-1 cells from Axin2+/lacZ and WT mice were isolated and the canonical Wnt signaling analyzed based on β-galactosidase (FDG+) activity. The percentage (A-C) and absolute number (B-D) FDG+ of each cell population were then calculated. n = 3 mice per group. *p≤0,01 (Mann-Whitney test). WT mice not carrying the reporter transgene (Axin2+/lacZ) were used to define the FDG^−^ population. In order to calculate the percentage or absolute number of FDG+ cells of each subset, FDG+ amount was subtracted from amount of FDG+ cells in the WT mice in order to correct differences in background staining.

The canonical Wnt signaling in B-1 cells was evaluated using the Axin2^+/lacZ^ Wnt-reporter mouse [28]. No difference in the amount of peritoneal B and T lymphocytes was observed in these mice compared to wild type strain. In both, B-1 cells correspond approximately to 60% of total lymphocytes (22% of total cells from peritoneal cavity (S3 Fig) in the peritoneal cavity. To assess activation of canonical Wnt signaling, Axin2 expression was measured by the β-galactosidase activity, using the fluorescein di β-D-galactopyranoside (FDG) as a fluorogenic β-galactosidase substrate. Axin2 activation was detected in less than 3% of B-1 cells (Fig 5A– 5B), indicating that the canonical Wnt signaling was not activated in these populations under steady state conditions (*ex vivo*). Despite of this, we observed activation of Wnt signaling in the B-1 cell precursor population (Fig 5C– 5D). In agreement with the literature, more mature stages of B cells have reduced Wnt signaling levels, while this pathway is active in the pre-proB stages (12).

### Wnt3a induce B-1 precursors to originated B-1a cells *in vitro*

**Fig 6:**
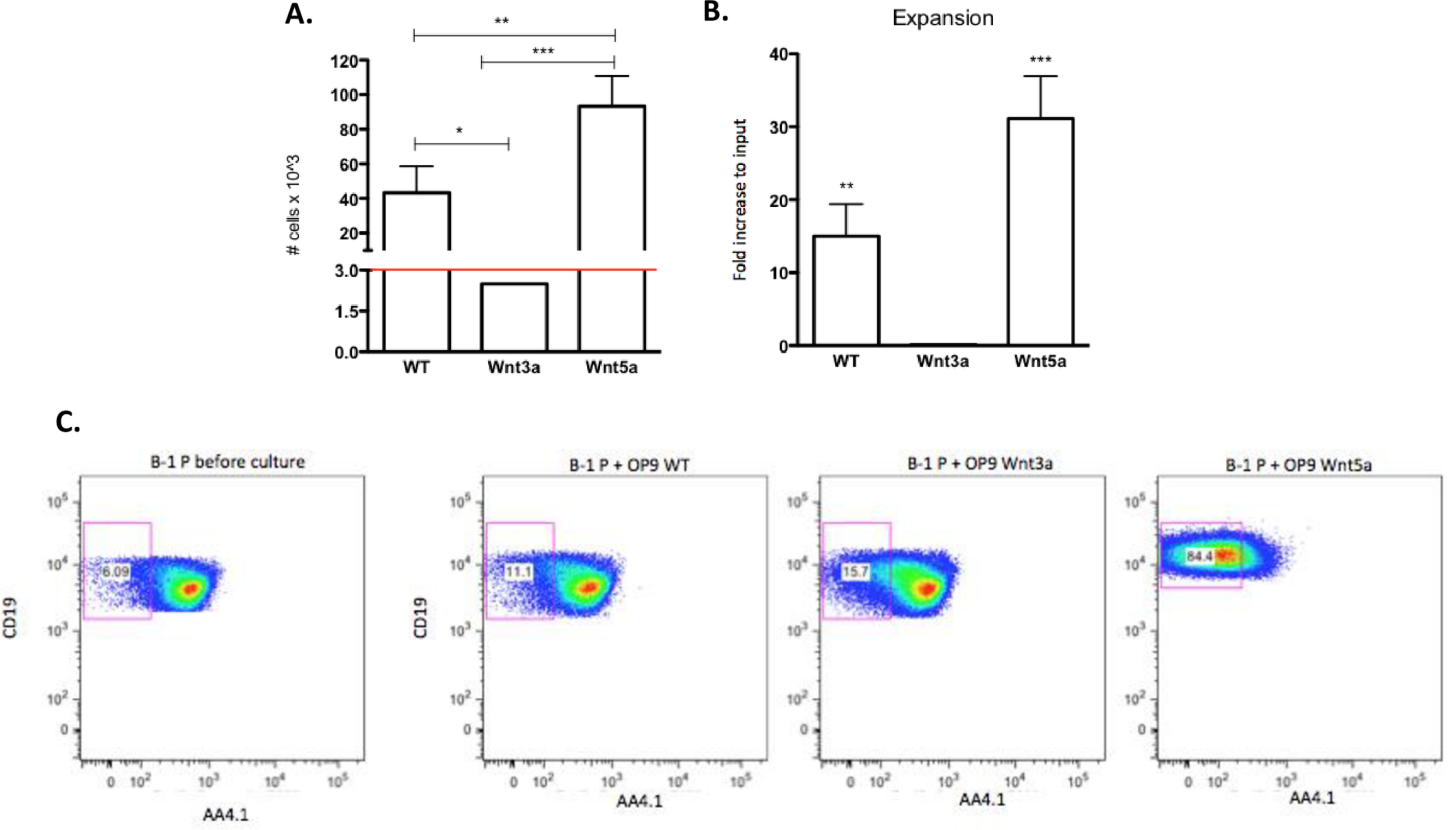
Wnt5a stimulates expansion of B-1P cells in vitro. B-1P cells were co-cultivated onto OP9-WT (WT), Wnt3a-transduced OP9 (Wnt3a) or Wnt5a-transduced OP9 (Wnt5a) layers. After 9 days, the number of B-1P cells in these cultures was analyzed. (A) Absolute number of B-1P cells co-cultivated with OP9-WT, OP9-Wnt3a and OP9-Wnt5a after 9 days of co-culture. The initial input of B-1P was 3x10^3^ cells, which is represented by red line. (B) Expansion of B-1 cell precursor population after 9 days in co-culture with OP9-WT, OP9-Wnt3a and OP9-Wnt5a. The graphs represents fold change increase in relation to input of cells. It was calculated as the number of B-1 cell precursors after 9 days in culture normalized by the initial input (3x10^3^ cells). (C) Dot plots of expression of CD19X AA4.1 by B-1P cells before culture and after 9 days in culture with OP9-WT, OP9-WNt3a, OP9-Wnt5a. Results are representative of 2 experiments performed in triplicate (n = 3). ***p<0,001, **p<0,01 and *p<0,05. (One way ANOVA).

To further investigate if the Wnt pathway is more activated in the early stages of B-1 cells, the activity was assessed in the B-1 cell precursors *in vitro* [29]. A co-culture experiment using Wnt-transduced OP9 cells, which constitutively express Wnt3a (OP9-Wnt3a) and Wnt5a (OP9-Wnt5a) [27] was performed. Considering the initial input (3x10^3^ cells), B-1P cells cultivated with OP9-WT increased almost 15x (43x10^3^ ± 8 cells) and on OP9-Wnt5a more than 40x (93x10^3^ ± 10 cells). The number of B-1P cells in the OP9-Wnt3a co-culture was roughly maintained at 2.5x10^3^ cells (Fig 6A and 6B). Furthermore, B-1P cells cultivated in the presence of OP9-Wnt5a mostly lost the expression of AA4.1 (Fig 6C). However, the expression of AA4.1 is sustained in B-1P cells cultivated in the presence of OP9-WT and OP9-Wnt3a. This data is suggestive that Wnt5a induced proliferation and differentiation in the B-1 precursors.

**Fig 7:**
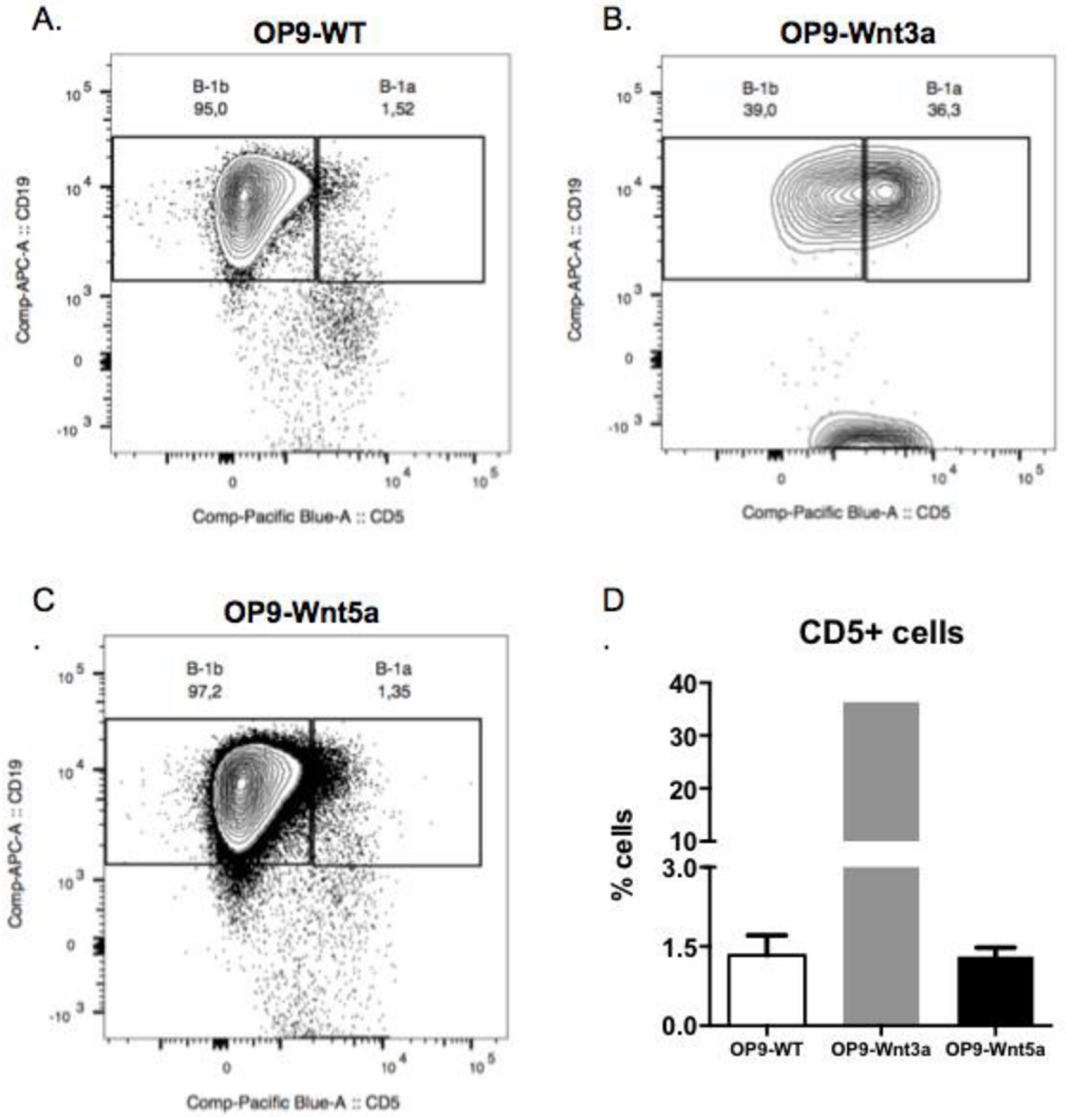
Generation of CD5+ B-1 cells from B-1P cells in vitro in the presence of Wnt3a. Analysis of CD19^+^CD5^-^ and CD19^+^CD5^+^ cells generated in the co-cultures of B-1P cells with OP9 stromal cells (A), Wnt3a-transduced OP9 cells (B) and Wnt5a-transduced OP9 cells (C). Dot plots CD19xCD5 were generated from AA4.1^-^cell population. The co-cultures were maintained for 9 days. (D) Percentage of CD19^+^CD5^-^ (B-1a) cells generated in each cell culture condition. Results are representative of 2 experiments performed in triplicate **p≤0,001 (Mann-Whitney test).

Additional flow cytometer analysis on B-1 precursors differentiated *in vitro* showed that only in the Wnt3a treated group CD5+ cells (B-1a) were generated, despite the lack of proliferation showed earlier (Fig 7). In order to separate B-1 cell precursor from a population of B-1 cells that could be generated in vitro, AA4.1+ cell population was excluded from the next analysis. From the AA4.1^-^ population, expression of CD19 and CD5 was analyzed to determine the frequency of B-1a (CD19^+^CD5^-^ cells) and B-1b (CD19^+^CD5^-^ cells). It is important to mention that all CD19^+^ cells were also IgM^+^ (*data not shown*). Based on this analysis, B-1 cell precursors co-cultivated with OP9-WT and OP9-Wnt5a showed only 1,5% of CD19^+^CD5^+^ cells (Fig 7A and 7C), while in the presence of Wnt3a 36% of CD19^+^ cells are also CD5+. However it is important to remember that the total number of cells in these latter cultures was reduced. Considering this, Wnt5a induced a pronounced expansion of B-1 cell precursors *in vitro*, which did not occur when these precursors were in the presence of Wnt3a (Fig 6). However, an interesting generation of CD5+ B-1 (B-1a) cells was observed in the presence of Wnt3a (Fig 7).

## Discussion

Here we report that the responsiveness of B-1 and B-1 precursors to Wnt ligands is different, which points to a role for the Wnt pathway in the regulation B-1 cell fate. As Malhotra et al [14] described on conventional B cells, at least two Wnt ligands can differentially regulate the B lymphopoiesis: Wnt3a and Wnt5. In summary, results described here demonstrated that: 1) The canonical Wnt pathway is not activated in B-1 cells in vivo, but it is in the B-1 progenitors (B-1P FDG+ cells were detected); 2) Wnt3a induces an increase in IL-7R expression followed by increased in B-1 cell proliferation *in vitro*; 3) Wnt5a induces an expansion of B-1 cell precursors, while Wnt3a is able to induce *de novo* B-1a generation in vitro.

The lower frequency of FDG^+^ B-1 cells is in accordance with data from literature, which show that the responsiveness to Wnt ligands diminishes along hematopoietic development, so it is predictable that canonical Wnt pathway could be detected in B-1 cell progenitors, but not later in B-1 cells.

Nevertheless, we demonstrated that both B-1 cells and B-1 cell precursors are responsive to Wnt ligands *in vitro*. B-1 cells proliferate in response to Wnt3a, and also augment the expression of IL7-R. Considering that IL7 is an important factor for proliferation of B cells, we could postulate that up regulation of expression of IL7R could be a mechanism related to an increment in the proliferation index of B-1 cells in the presence of Wnt3a. We also speculate that Wnt3a could be an important factor for the maintenance of self-renewal of B-1 cell population in the peritoneal cavity. Unfortunately, no data was found in the literature about the expression of Wnt ligands in the peritoneum in normal and health conditions that could support this hypothesis. Conversely, Wnt5a stimulus did not modify the B-1 cell proliferation activity in vitro. It is widely believed that Wnt5a could antagonize the canonical Wnt pathway. Liang et al [30] demonstrated that Wnt5a inhibits the pro-B cell response to IL7, via noncanonical Wnt/Ca^+2^ pathway. We here showed that in a Wnt3a enriched milieu, proliferation of B-1 cells could be favored by augmented IL7R expression, in accordance with the notion that Wnt5a signals diminish the responsiveness to IL7R.

Canonical Wnt signaling in B-1 precursors promotes differentiation into B-1a cells in vitro, but also is permissive for B-1b cell development. Conversely, a small B-1a cell population is observed in a B-1P+OP9-Wnt5a culture. As demonstrated by Famili et al. [27], the OP9-Wnt3a cell line has over 1,000 fold higher expression of Wnt3a than OP9-WT (non-transduced). It is important to mention that the timing and concentration of ligand exposure is a determinant to reflect the effect of Wnt pathway on the cell behaviour. The generation of B-1a cells could be a result of differentiation of B-1 precursors into B1a cells, but also it is possible that Wnt3a exposure in our co-cultures expanded rare and preexisting cells B-1a cells that could not be detected at the start of the culture.

We observed a marked expansion of B1-P progenitors in the presence of Wnt5a, accompanied by loss of AA4.1 expression. Previous reports demonstrated that murine CD19^+^ and human CD34^+^CD38^-^ fail to differentiated into CD19^+^ B cell lineage on OP9-Wnt3a co-cultures, but were favored on OP9-Wnt5a and OP9-Dkk1 ones [14, 31]. It could be considered that the bone marrow microenvironment is rich in Wnt5a [16] and it could support the B cell development. Reya et al [16] also demonstrated that bone marrow stromal cells expressed Wnt5a, but not Wnt10b and Wnt3a, which would support B cell development. In this context, bone-marrow could sustain the maintenance of B-1 cell precursor by expression of Wnt5a.

Despite of the reduced overall number of cells, we demonstrated that only in the presence of Wnt3a, B-1 cell precursors give rise to B-1a cells *in vitro*. Yoshimoto et al [3] described the emergence of B-1 progenitors before the HSC stage, at embryonic day 9.0–9.5 from yolk sac and intraembryonic para-aortic splanchnopleura (PSp) tissues. Recently the Herzenberg group showed that fetal liver CD150^-,^ but not CD150^+^ LT-HSCs were able to reconstitute the B-1a cell population [6], reinforcing the separate origins of B-1 cell progenitors. Interestingly, it has been demonstrated that the Wnt signature of stromal cells in the adult and fetal bone marrow-derived mesenchymal stromal cells are different. Therefore, Wnt ligands in the fetal liver and bone marrow could result in differential responsiveness of the precursors, and the balance of Wnt ligands could influence B-1 population expansion and govern the B-1 cell precursor development.

Based on this, we postulate that the canonical Wnt pathway could be important in the development of B-1 cell precursors, and somehow could interfere in the proliferation of B-1 cells in response to IL7. It could be suggested that Wnt5a regulates the expansion of B-1 progenitors in the adult bone marrow, while Wnt3a could interfere in the generation of B-1a cells in the bone marrow and also in the proliferation of the B-1 cells, perhaps by controlling the self-renewal activity. It is intriguing to conceive that HSCs must have an intrinsic control to maintain the steady-state hematopoiesis during lifetime, and be able to promote reconstitution of cell populations after an injury. Similar mechanisms may operate for B-1 cells. Moreover, a loss of control in the self-renewal or differentiation process could lead to generation of malignant cells which for B-1 cells could lead to B-CLL, the malignant counterpart of normal B-1 cells. It remains unclear whether Wnt signaling is indispensible for B-1 cell development. However our results shed some light on this issue and show how members of this pathway could determine the development and maintenance of B-1 cells. Whether such regulation of B-1 cell lineage fate decisions by Wnt signaling also occurs *in vivo*, waits for complicated loss-of-function models that are currently unavailable, especially for the non-canonical pathway. Given the cross talk and redundancy in the Wnt pathway, this question is a challenging one to address *in vivo*.

## Supporting information

S1 Fig Characterization of peritoneal B-1 cells. First, the lymphocytes were gated (A) and doublet excluded (data not shown). The B cells populations were defined by the CD19 and CD23 expression (B), in which B-1 cells presented CD19^+^CD23^-^ and B-2 cells, CD19^+^CD23^+^.(C) Expression of CD5 define B-1a and B-1b cell population.
(TIFF)

S2 Fig Characterization of bone-marrow derived B-1 cell precursors. To analyze the populations, first the lymphocyte population was gated (A), doublets excluded (data not shown) and the Lin- and CD93^+^(Early B^+^) population was selected (B). Based on the CD19 and B220 expression, the B-1P (CD19^+^B220^lo/neg^), Pre/Pro-B (CD19^-^B220^+^) and Pro-B (CD19^+^B220^+^) populations were determined (C).
(TIFF)

S3 Fig Analysis of peritoneal B-1 cells, B-2 cells and T cells from Axin2+/lacZ mice and WT mice reveals that cell population size is similar in both mice. Percentage (A) and absolute number of each cell.
(TIFF)

## References

[1] Montecino-Rodriguez E, Leathers H, Dorshkind K. Identification of a B-1 B cell-specified progenitor. Nat Immunol. 2006;7(3):293–301. Epub 2006/01/24. doi: 10.1038/ni1301 . 16429139 10.1038/ni1301

[2] Dorshkind K, Montecino-Rodriguez E. Fetal B-cell lymphopoiesis and the emergence of B-1-cell potential. Nat Rev Immunol. 2007;7(3):213–9. doi: 10.1038/nri2019 . 17318232 10.1038/nri2019

[3] Yoshimoto M, Montecino-Rodriguez E, Ferkowicz MJ, Porayette P, Shelley WC, Conway SJ, et al Embryonic day 9 yolk sac and intra-embryonic hemogenic endothelium independently generate a B-1 and marginal zone progenitor lacking B-2 potential. Proc Natl Acad Sci U S A. 2011;108(4):1468–73. doi: 10.1073/pnas.1015841108 ; PubMed Central PMCID: PMCPMC3029764. 21209332 10.1073/pnas.1015841108 PMC3029764

[4] Montecino-Rodriguez E, Dorshkind K. B-1 B cell development in the fetus and adult. Immunity. 2012;36(1):13–21. doi: 10.1016/j.immuni.2011.11.017 ; PubMed Central PMCID: PMCPMC3269035. 22284417 10.1016/j.immuni.2011.11.017 PMC3269035

[5] Kantor AB, Herzenberg LA. Origin of murine B cell lineages. Annual review of immunology. 1993;11:501–38. Epub 1993/01/01. doi: 10.1146/annurev.iy.11.040193.002441 . 8476571 10.1146/annurev.iy.11.040193.002441

[6] Ghosn EE, Waters J, Phillips M, Yamamoto R, Long BR, Yang Y, et al Fetal Hematopoietic Stem Cell Transplantation Fails to Fully Regenerate the B-Lymphocyte Compartment. Stem Cell Reports. 2016;6(1):137–49. doi: 10.1016/j.stemcr.2015.11.011 . 26724903 10.1016/j.stemcr.2015.11.011 PMC4720028

[7] Nusse R. Wnt signaling in disease and in development. Cell Res. 2005;15(1):28–32. doi: 10.1038/sj.cr.7290260 . 15686623 10.1038/sj.cr.7290260

[8] Staal FJ, Clevers HC. WNT signalling and haematopoiesis: a WNT-WNT situation. Nat Rev Immunol. 2005;5(1):21–30. doi: 10.1038/nri1529 . 15630426 10.1038/nri1529

[9] Staal FJ, Luis TC, Tiemessen MM. WNT signalling in the immune system: WNT is spreading its wings. Nat Rev Immunol. 2008;8(8):581–93. doi: 10.1038/nri2360 . 18617885 10.1038/nri2360

[10] Reya T, Clevers H. Wnt signalling in stem cells and cancer. Nature. 2005;434(7035):843–50. doi: 10.1038/nature03319 . 15829953 10.1038/nature03319

[11] Reya T, Duncan AW, Ailles L, Domen J, Scherer DC, Willert K, et al A role for Wnt signalling in self-renewal of haematopoietic stem cells. Nature. 2003;423(6938):409–14. doi: 10.1038/nature01593 . 12717450 10.1038/nature01593

[12] Luis TC, Naber BA, Roozen PP, Brugman MH, de Haas EF, Ghazvini M, et al Canonical wnt signaling regulates hematopoiesis in a dosage-dependent fashion. Cell Stem Cell. 2011;9(4):345–56. doi: 10.1016/j.stem.2011.07.017 . 21982234 10.1016/j.stem.2011.07.017

[13] Luis TC, Ichii M, Brugman MH, Kincade P, Staal FJ. Wnt signaling strength regulates normal hematopoiesis and its deregulation is involved in leukemia development. Leukemia. 2012;26(3):414–21. doi: 10.1038/leu.2011.387 ; PubMed Central PMCID: PMCPMC3378318. 22173215 10.1038/leu.2011.387 PMC3378318

[14] Malhotra S, Baba Y, Garrett KP, Staal FJ, Gerstein R, Kincade PW. Contrasting responses of lymphoid progenitors to canonical and noncanonical Wnt signals. J Immunol. 2008;181(6):3955–64. ; PubMed Central PMCID: PMCPMC2562264. 18768850 10.4049/jimmunol.181.6.3955 PMC2562264

[15] Baba Y, Garrett KP, Kincade PW. Constitutively active beta-catenin confers multilineage differentiation potential on lymphoid and myeloid progenitors. Immunity. 2005;23(6):599–609. doi: 10.1016/j.immuni.2005.10.009 ; PubMed Central PMCID: PMCPMC1850237. 16356858 10.1016/j.immuni.2005.10.009 PMC1850237

[16] Reya T, O'Riordan M, Okamura R, Devaney E, Willert K, Nusse R, et al Wnt signaling regulates B lymphocyte proliferation through a LEF-1 dependent mechanism. Immunity. 2000;13(1):15–24. . 10933391 10.1016/s1074-7613(00)00004-2

[17] Reya T, Okamura R, Grosschedl R. Control of lymphocyte differentiation by the LEF-1/TCF family of transcription factors. Cold Spring Harb Symp Quant Biol. 1999;64:133–40. . 11232278 10.1101/sqb.1999.64.133

[18] Yu Q, Quinn WJ, Salay T, Crowley JE, Cancro MP, Sen JM. Role of beta-catenin in B cell development and function. J Immunol. 2008;181(6):3777–83. ; PubMed Central PMCID: PMCPMC2575415. 18768830 10.4049/jimmunol.181.6.3777 PMC2575415

[19] Novo MC, Osugui L, dos Reis VO, Longo-Maugéri IM, Mariano M, Popi AF. Blockage of Wnt/β-catenin signaling by quercetin reduces survival and proliferation of B-1 cells in vitro. Immunobiology. 2015;220(1):60–7. doi: 10.1016/j.imbio.2014.09.001 . 25245014 10.1016/j.imbio.2014.09.001

[20] Popi AF, Motta FL, Mortara RA, Schenkman S, Lopes JD, Mariano M. Co-ordinated expression of lymphoid and myeloid specific transcription factors during B-1b cell differentiation into mononuclear phagocytes in vitro. Immunology. 2009;126(1):114–22. Epub 2008/08/20. doi: 10.1111/j.1365-2567.2008.02883.x ; PubMed Central PMCID: PMCPmc2632701. 18710404 10.1111/j.1365-2567.2008.02883.x PMC2632701

[21] Almeida SR, Aroeira LS, Frymuller E, Dias MA, Bogsan CS, Lopes JD, et al Mouse B-1 cell-derived mononuclear phagocyte, a novel cellular component of acute non-specific inflammatory exudate. International immunology. 2001;13(9):1193–201. Epub 2001/08/30. . 11526100 10.1093/intimm/13.9.1193

[22] Popi AF, Osugui L, Perez KR, Longo-Maugeri IM, Mariano M. Could a B-1 cell derived phagocyte "be one" of the peritoneal macrophages during LPS-driven inflammation? PLoS One. 2012;7(3):e34570 Epub 2012/04/06. doi: 10.1371/journal.pone.0034570 ; PubMed Central PMCID: PMCPmc3316698. 22479646 10.1371/journal.pone.0034570 PMC3316698

[23] Schmittgen TD, Livak KJ. Analyzing real-time PCR data by the comparative C(T) method. Nat Protoc. 2008;3(6):1101–8. . 18546601 10.1038/nprot.2008.73

[24] Vandesompele J, De Preter K, Pattyn F, Poppe B, Van Roy N, De Paepe A, et al Accurate normalization of real-time quantitative RT-PCR data by geometric averaging of multiple internal control genes. Genome Biol. 2002;3(7):RESEARCH0034. ; PubMed Central PMCID: PMCPMC126239. 12184808 10.1186/gb-2002-3-7-research0034 PMC126239

[25] Nolan GP, Fiering S, Nicolas JF, Herzenberg LA. Fluorescence-activated cell analysis and sorting of viable mammalian cells based on beta-D-galactosidase activity after transduction of Escherichia coli lacZ. Proc Natl Acad Sci U S A. 1988;85(8):2603–7. ; PubMed Central PMCID: PMCPMC280046. 3128790 10.1073/pnas.85.8.2603 PMC280046

[26] Fiering SN, Roederer M, Nolan GP, Micklem DR, Parks DR, Herzenberg LA. Improved FACS-Gal: flow cytometric analysis and sorting of viable eukaryotic cells expressing reporter gene constructs. Cytometry. 1991;12(4):291–301. doi: 10.1002/cyto.990120402 . 1905992 10.1002/cyto.990120402

[27] Famili F, Naber BA, Vloemans S, de Haas EF, Tiemessen MM, Staal FJ. Discrete roles of canonical and non-canonical Wnt signaling in hematopoiesis and lymphopoiesis. Cell Death Dis. 2015;6:e1981 doi: 10.1038/cddis.2015.326 ; PubMed Central PMCID: PMCPMC4670932. 26583322 10.1038/cddis.2015.326 PMC4670932

[28] Jho EH, Zhang T, Domon C, Joo CK, Freund JN, Costantini F. Wnt/beta-catenin/Tcf signaling induces the transcription of Axin2, a negative regulator of the signaling pathway. Mol Cell Biol. 2002;22(4):1172–83. doi: 10.1128/MCB.22.4.1172-1183.2002 ; PubMed Central PMCID: PMCPMC134648. 11809808 10.1128/MCB.22.4.1172-1183.2002 PMC134648

[29] Ghosn EE, Sadate-Ngatchou P, Yang Y, Herzenberg LA, Herzenberg LA. Distinct progenitors for B-1 and B-2 cells are present in adult mouse spleen. Proc Natl Acad Sci U S A. 2011;108(7):2879–84. doi: 10.1073/pnas.1019764108 ; PubMed Central PMCID: PMCPMC3041118. 21282663 10.1073/pnas.1019764108 PMC3041118

[30] Liang H, Chen Q, Coles AH, Anderson SJ, Pihan G, Bradley A, et al Wnt5a inhibits B cell proliferation and functions as a tumor suppressor in hematopoietic tissue. Cancer Cell. 2003;4(5):349–60. . 14667502 10.1016/s1535-6108(03)00268-x

[31] Ichii M, Frank MB, Iozzo RV, Kincade PW. The canonical Wnt pathway shapes niches supportive of hematopoietic stem/progenitor cells. Blood. 2012;119(7):1683–92. doi: 10.1182/blood-2011-07-369199 ; PubMed Central PMCID: PMCPMC3286346. 22117039 10.1182/blood-2011-07-369199 PMC3286346

