# Supplementary figures and images for: B-1 cells and B-1 cell precursors prompt different responses to Wnt signaling
Source: PLoS One. 2018 Jun 21;13(6):e0199332. doi: 10.1371/journal.pone.0199332 (PMC6013157; doi:10.1371/journal.pone.0199332)

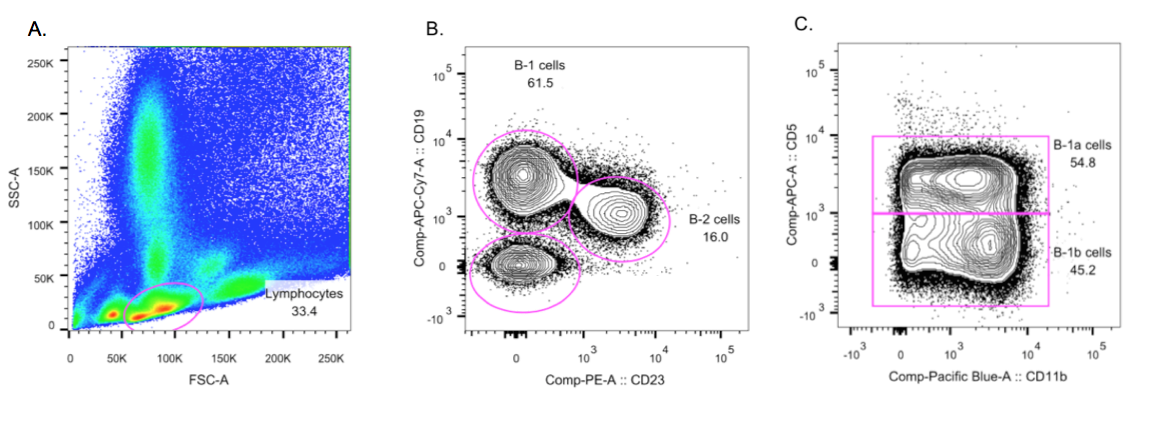

Supplement: S1 Fig — First, the lymphocytes were gated (A) and doublet excluded (data not shown). The B cells populations were defined by the CD19 and CD23 expression (B), in which B-1 cells presented CD19+CD23- and B-2 cells, CD19+CD23+.(C) Expression of CD5 define B-1a and B-1b cell population. (TIFF) [file pone.0199332.s001.tiff]

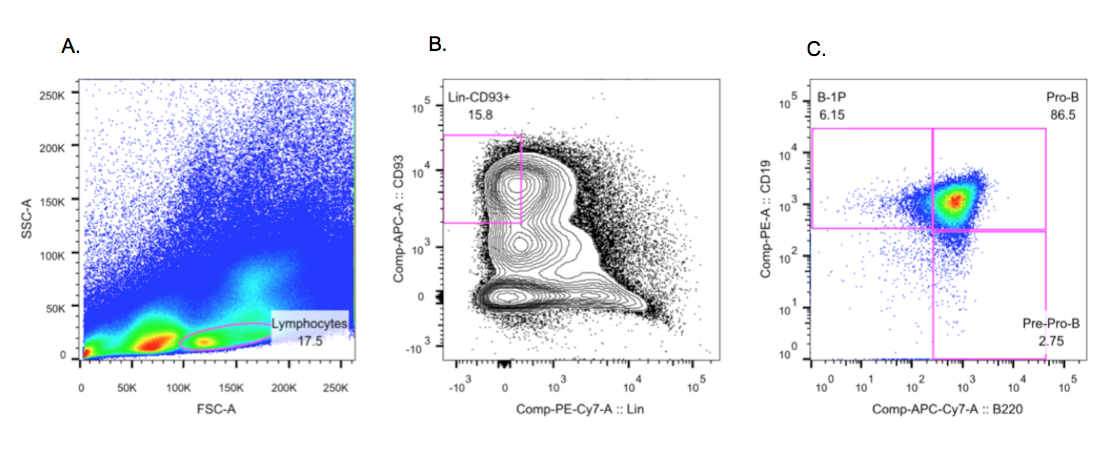

Supplement: S2 Fig — To analyze the populations, first the lymphocyte population was gated (A), doublets excluded (data not shown) and the Lin- and CD93+(Early B+) population was selected (B). Based on the CD19 and B220 expression, the B-1P (CD19+B220lo/neg), Pre/Pro-B (CD19-B220+) and Pro-B (CD19+B220+) populations were determined (C). (TIFF) [file pone.0199332.s002.tiff]

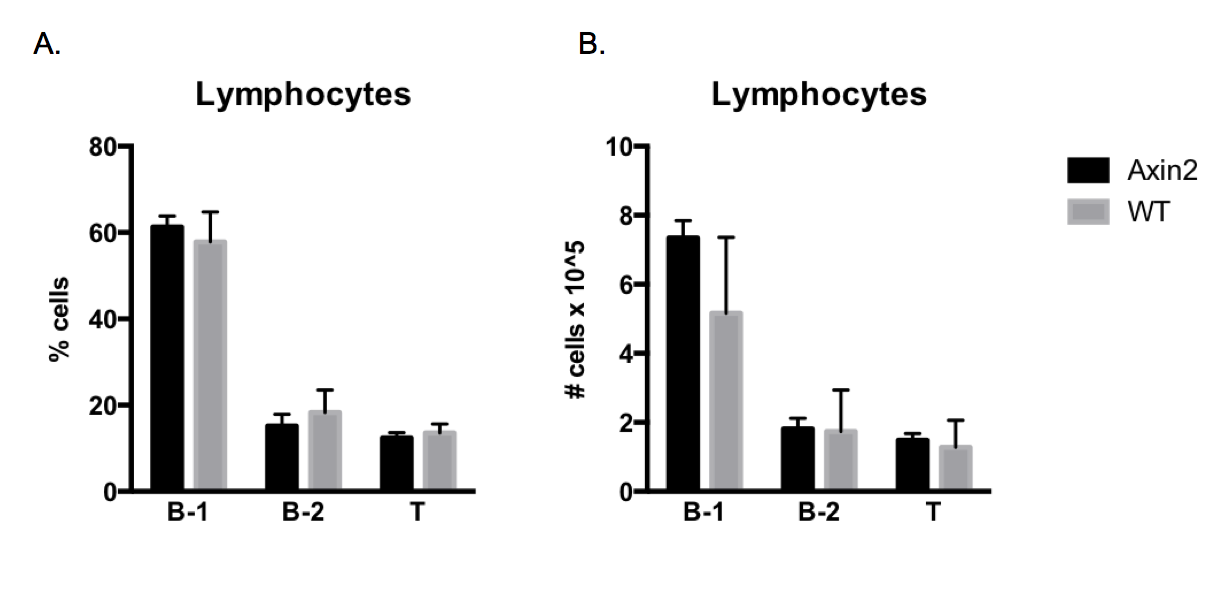

Supplement: S3 Fig — Percentage (A) and absolute number of each cell. (TIFF) [file pone.0199332.s003.tiff]
